# Relation between HIV status, risky sexual behavior, and mental health in an MSM sample from three Chilean cities

**DOI:** 10.26633/RPSP.2017.4

**Published:** 2017-02-08

**Authors:** Fabiola Gómez, Jaime Barrientos, Manuel Càrdenas

**Affiliations:** 1 Escuela de Psicología Pontificia Universidad Católica de Chile Santiago Chile Escuela de Psicología, Pontificia Universidad Católica de Chile, Santiago, Chile.; 2 Escuela de Psicología Universidad de Santiago de Chile Santiago Chile Escuela de Psicología, Universidad de Santiago de Chile, Santiago, Chile.; 3 Escuela de Psicología Universidad de Valparaíso, Chile Escuela de Psicología, Universidad de Valparaíso, Chile.

**Keywords:** HIV infection; mental health, sexual behavior, Chile, Latin America., Infecciones por VIH, salud mental, conducta sexual, Chile; América Latina

## Abstract

**Objective.:**

To explore the association among HIV status; negative psychological symptoms (anxiety, depression, and hostility); and risky sexual behaviors (multiple sexual partners and unprotected sexual intercourse) in a Chilean sample of men who have sex with men (MSM).

**Methods.:**

*This study had a cross-sectional design and a sample of 325 MSM whose ages ranged from 18 to 64 years (mean: 30.8; standard deviation: 9.8). Association tests (chisquared) and group mean comparisons (Student’s* t*-tests and* F*-tests) were performed.*

**Results.:**

No statistically significant differences were found for condom use or for the number of sexual partners between HIV-positive men and those who are not infected. In both groups, about 50% reported sexual encounters without condom use in the past six months. There were statistically significant differences in symptoms associated with depression between the two groups.

**Conclusions.:**

These results reveal the need to strengthen messages about the importance of condom use, as the only way to prevent HIV, and as a means of preventing HIV infection and reinfection, in national prevention and self-care programs for sexually active subjects. More studies are needed in Latin America to advance HIV prevention efforts for the MSM population. The data generated by this study can be used to inform the development of HIV prevention programming strategies and interventions targeting the MSM population in Latin America.

## RESUMEN

**Objetivo.:**

Explorar la relación entre el estado con respecto a la infección por el VIH, los síntomas psicológicos negativos (ansiedad, depresión y hostilidad) y el comportamiento sexual arriesgado (varios compañeros sexuales y relaciones sexuales sin protección) en una muestra de hombres que tienen relaciones sexuales con otros hombres (HSH) de Chile.

**Métodos.:**

Este estudio tuvo un diseño transversal y una muestra de 325 HSH de 18 a 64 años (media: 30,8; desviación estándar: 9,8). Se efectuaron pruebas de asociación (ji al cuadrado) y comparaciones entre las medias grupales (prueba de la t de Student y prueba F).

**Resultados.:**

No se observaron diferencias estadísticamente significativas respecto del uso de preservativos o del número de compañeros sexuales entre los hombres seropositivos al VIH y los no infectados. En los dos grupos, más del 50 % refirió que había tenido algún encuentro sexual sin protección en los seis meses anteriores. Se observaron diferencias estadísticamente significativas entre los dos grupos en cuanto a los síntomas asociados a la depresión.

**Conclusiones.:**

Estos resultados muestran la necesidad de fortalecer los mensajes acerca de la importancia del uso de preservativos como la única manera de prevenir el VIH y como un medio de prevenir la infección y la reinfección por este virus, en los programas nacionales de prevención y autocuidado destinados a las personas sexualmente activas. Es necesario efectuar más estudios en América Latina para impulsar las medidas de prevención de la infección por el VIH orientadas a la población de HSH. Los datos generados por este estudio pueden servir para formular estrategias programáticas de prevención e intervenciones dirigidas a esta población en América Latina.

Studies on HIV worldwide reported by the Joint United Nations Programme on HIV/AIDS (UNAIDS) indicate that the rate of HIV infection has remained relatively stable, but the number of subjects dying from HIV-related diseases has decreased (1). This effect may be mainly due to the availability of antiretroviral therapy (ART).

In Chile, the HIV epidemic mainly affects people 20–39 years old (mostly men), and sexual intercourse is the main route of exposure to the virus. According to data from the Ministry of Health (*Ministerio de Salud*, MINSAL) (2), the regions with the highest HIV/AIDS rates are Arica and Parinacota, the Metropolitan Region, Tarapacá, and Valparaíso.

Prevalence estimates by MINSAL reveal that the group most affected by HIV/AIDS is the MSM (men who have sex with men) population. A recent and nonrecurring HIV prevalence study with a sample from a Chilean MSM population showed that 21.8% of respondents were HIV-positive men (3).

To establish criteria favoring HIV/AIDS intervention and prevention plans, several studies have addressed risky sexual behavior in MSM. Some of these behaviors are having multiple sexual partners, inconsistent or no condom use, and anal sexual relations (4–6). All of these behaviors can increase the probability of HIV infection. In addition, the MSM population has been associated with greater prevalence of depressive symptomatology, negative mood, anxiety, and loneliness (7, 8). These symptoms, in turn, have been related to risky sexual behavior, although results about this relationship may not be conclusive (9, 10).

Other studies report a relationship between HIV infection and psychological symptoms, such as anxiety and depression (11–13), as well as risky sexual behaviors, such as unprotected sex (11, 14–16).

In Chile, no studies have been carried out on the relationship between the MSM group, HIV status, and risky sexual behavior that simultaneously consider other associated variables (e.g., psychological symptoms). Therefore, this study explores the association among HIV status; negative psychological symptoms (anxiety, depression, and hostility); and risky sexual behaviors (multiple sexual partners and unprotected sexual intercourse) in a Chilean MSM sample. The findings from this research could be used to help build a comprehensive framework for this issue and contribute to developing future HIV intervention and prevention programs specifically directed to one of the most affected groups.

## MATERIALS AND METHODS

### Study design, sample, and recruiting procedure

This cross-sectional study focused on MSM—a difficult-to-reach or “hidden” population (17). For this reason, the study used respondent-driven sampling (RDS) (18). The profile of the target group was defined, and socio-demographically diverse initial participants (seeds) meeting the criteria were selected. Three seeds were non-randomly selected, with input from key informants in each city, as the starting points for recruitment. The criteria for selecting the seeds were as follows: MSM with many network connections who lived in Arica, Santiago, or Valparaíso; fell into one of three age groups (18–29, 30–44, and 45 years or more, with each of the three seeds representing one of the three groups); and provided written informed consent.

Three cities were chosen for data collection (Arica, Santiago, and Valparaíso) based on their high HIV/AIDS prevalence, according to official statistics (2).

The following question was used to assess MSM network connections: “How many gay men or MSM do you know who know you and live in this city?” After completion of the survey, each seed was instructed to invite three MSM who met the eligibility criteria to participate in the study. If the seed knew a potential participant, the research team contacted him by telephone. The new seed was then provided with a brief description of the project. This process was repeated until the sample size was reached (six waves).

The survey instrument was administered face-to-face and took about 45 minutes to complete. Participants were informed about the study objectives and asked to sign an acceptance letter ensuring data confidentiality. The interviewers signed a letter to each participant guaranteeing the confidentiality of the data collected.

The study was approved by the Universidad Católica del Norte Ethics Commission (Antofagasta) and the National Fund for Scientific and Technological Development (*Fondo Nacional de Desarrollo Científico y Tecnológico*, FONDECYT) (Santiago).

### Measures

**Socio-demographic characteristics. **Participants reported their age and city of residence. Age was recategorized into three age groups (18–29, 30–44, and 45 years or more). Participants also reported their highest education level. Response options ranged from “incomplete primary education” to “graduate degree(s)” (Master’s degree, Doctorate, or equivalent). This variable was recategorized into two levels of education—“without tertiary studies” and “with tertiary studies” (technical school or college).

**Risky sexual behavior and HIV status. **This section included questions specifically designed for this study. Participants were asked about the number of sexual partners they had had in the past six months. Responses options included “none,” “only one,” “two to four,” “five to nine,” and “10 or more.” Participants were also asked about the number of times they had had sexual relations without condom use in the past six months (“none,” “only once,” “twice,” “three to five times,” and “six or more times”); whether or not they had taken the HIV test (“Yes” / “No”); and, if so, their status (“HIV-positive” or “HIV-negative”).

**Symptomatology.** This study used the Spanish version of the Symptom Checklist-90-Revised (SCL-90-R®) (*Inventario de Síntomas de Derogatis Revisado*) (Derogatis, 1979), adapted to the Chilean population (19). The Spanish version includes nine dimensions (symptoms). The survey instructs the respondent to indicate which symptoms had troubled or bothered him/her over the past three weeks, and to what degree. For this application, Likert-type response options ranging from 1 (“nothing at all” [bothered/troubled him/her]) to 6 ([was bothered/troubled] “quite a lot; extremely often”) were used. A higher score indicated greater symptomatology. In this study, only three of the nine dimensions were included in the survey (anxiety, depression, and hostility, the most widely reported symptomatology in mental health studies on the homosexual population (20)). The section on depression included 13 survey items (e.g., “Think about whether you have had little energy or been in a bad mood in the past few weeks”); the anxiety section included 10 items (e.g., “Think about whether you have felt afraid of something in the past few weeks”); and the hostility section included six items (e.g., “Think about whether you have felt like throwing things in the past few weeks”).

### Statistical analyses

Univariate and bivariate statistical analyses were conducted using SPSS version 20 software (IBM SPSS Inc., Chicago, Illinois, United States). Association tests (chi-squared) were used to analyze the relationship between HIV status and risky sexual behavior, and group mean comparisons (Student’s *t*-tests and *F*-tests) were used to analyze the relationship between reported symptoms and risky sexual behavior. Effect sizes (*w*, *d*, and *f*)4 were calculated for all statistically significant tests using G*Power 3.1 (Heinrich Heine University, Düsseldorf, Germany) (21). Data were analyzed using the RDS Analysis Tool, version 7.1.3 (RDSAT, Cornell University, Ithaca, New York, United States).

## RESULTS

### Sample

The sample included 325 men whose ages ranged from 18 to 64 years (mean (M): 30.8; standard deviation (SD): 9.8). Participants lived in Santiago (49.5%), Arica (24.6%), and Valparaíso (24.0%). The sample had a high level of education, as shown in Table 1. More than 60% had tertiary education studies (technical school or college).

### Risky sexual behavior and HIV status

A total of 96.6% of the study participants had been tested knew their HIV serological status, and 17.8% were HIV-positive. A total of 62.7% of participants reported having one to four sexual partners in the past six months, and 50.5% said they had always used a condom in the past six months (Table 2).

In analyzing the relationship between HIV status and risky sexual behavior, no statistically significant differences were found for condom use (*X*2(4) = 1.84; *P* = 0.77; *w* = 0.28) or the number of sexual partners (*X*2(4) = 2.23; *P* = 0.69; *w* = 0.23). In both groups (HIV-positive and HIV-negative), more than 40% reported one or no sexual partners in the past six months and about 50% reported sexual encounters without condom use in the past six months.

### Symptomatology and mean differences

In general, the levels of symptomatology reported (SCL-90-R® scores) were below the theoretical midpoint (3.5 on a six-point scale). The highest level of symptoms was reported for depression (M: 2.29; SD: 1.01), followed by anxiety (M: 2.09; SD: 1.03), and hostility (M: 2.05; SD: 1.15). Moreover, as expected, there were statistically significant differences in symptoms associated with depression between HIV-positive MSM and HIV-negative MSM (*t*(305) = 2.32; *P* = 0.02; *d* = 0.34). HIV-positive MSM reported higher levels of depression symptoms (M: 2.57; SD: 1.02) than HIV-negative MSM (M: 2.23; SD: 0.99). No differences were found for anxiety (*t*(305): 1.41; *P*: 0.15; *d*: 0.20) or hostility (*t*(304): 0.09; *P*: 0.85; *d*: 0.04).

**TABLE 1.** Sociodemographic characteristics of participants in study on HIV status, risky sexual behavior, and mental health in men who have sex with men (MSM) in three cities, Chile, 2010

Characteristic	No.^a^	%
City		
Arica	78	24.6
Valparaíso	80	24.0
Santiago	161	49.5
Age group (years)		
18–29	172	52.9
30–44	111	34.2
45 or more	42	12.9
HIV serological status		
HIV+	58	17.8
HIV–	256	78.8
Education level		
With tertiary studies	205	63.1
Without tertiary studies	118	36.3

***Source:*** Prepared by the authors based on the study results.

a Total sample may differ by variable due to missing data.

**TABLE 2.** Sexual behavior in past six months among participants in study on HIV status, risky sexual behavior, and mental health in men who have sex with men (MSM) in three cities, Chile, 2010^a^

Sexual behavior	No. (%)	No. (%)	
		HIV+	HIV–
Number of sexual partners			
None	29 (8.9)	5 (8.6)	24 (9.4)
1	107 (32.9)	20 (34.5)	85 (33.2)
2–4	97 (29.8)	13 (22.4)	79 (30.9)
5–9	47 (14.5)	9 (15.5)	34 (13.3)
10 or more	37 (11.4)	9 (15.5)	28 (10.9)
Number of sexual encounters without condom use			
None	164 (50.5)	31 (53.4)	126 (49.2)
Once	49 (15.1)	8 (13.8)	39 (15.2)
Twice	38 (11.7)	7 (12.1)	30 (11.7)
3–5	27 (8.3)	5 (8.6)	21 (8.2)
6 or more	38 (11.7)	4 (6.9)	34 (13.3)

***Source:*** Prepared by the authors based on the study results.

a Total sample may differ by variable due to missing data.

Statistically significant differences were found for anxiety between participants reporting one sexual partner or five to nine partners and those reporting 10 or more (Table 3), with subjects with 10 or more sexual partners in the past six months reporting higher levels of symptoms.

A statistically significant relationship was found between symptoms associated with hostility and the number of sexual encounters without condom use. A greater number of sexual relations without condom use in the past six months indicated higher levels of hostility (Table 4).

**TABLE 3.** One-way analysis of variance: summary table for relation between level of SCL-90-R®^a^ symptoms (score) and number of sexual partners in past six months for participants in study on HIV status, risky sexual behavior, and mental health in men who have sex with men (MSM) in three cities, Chile, 2010^b^,^c^

SCL-90-R®symptom	No. of sexual partners	No. of participants	Score		F-test	P^f^	df^g^	f^h^
			M^d^	SD^e^
Depression	None	29	2.38	1.01	1.27	0.28	4 – 310	0.13
	1	105	2.21	1.03				
	2–4	97	2.22	0.94				
	5–9	47	2.26	0.93				
	10 or more	37	2.60	1.12				
Anxiety	None	29	2.34	1.18	3.19	0.01^i^	4 – 310	0.20
	1^i^	105	1.95	1.00				
	2–4	97	2.04	0.94				
	5–9^i^	47	1.94	0.89				
	10 or morei	37	2.54	1.19				
Hostility	None	29	2.05	1.19	0.38	0.82	4 – 310	0.08
	1	105	2.03	1.24				
	2–4	97	1.95	1.12				
	5–9	47	2.17	1.11				
	10 or more	37	2.14	1.01				

***Source:*** Prepared by the authors based on the study results.

a Symptom Checklist-90-Revised (Derogatis, 1979).

b Possible scores range from 1 to 6; higher scores indicate greater symptom report.

c Total sample may differ by variable due to missing data.

d Mean.

e Standard deviation.

f *P* value for the *F*-test.

g Degrees of freedom.

h Effect size index *f*.

i Significance level *P* < 0.05.

**TABLE 4.** One-way analysis of variance: summary table for relation between level of SCL-90-R®^a^ symptoms (score) and number of sexual encounters without condom use in past six months for participants in study on HIV status, risky sexual behavior, and mental health in men who have sex with men (MSM) in three cities, Chile, 2010^b^,^c^

SCL-90-R®symptom	No. of sexual encounters without condom	No. of participants	Score		F-test	P^f^	df^g^	f^h^
			M^d^	SD^e^
Depression	None	162	2.25	1.03	1.49	0.21	4 – 309	0.14
	Once	49	2.38	1.01				
Twice	38	2.24	0.88				
3–5	27	2.66	1.08				
6 or more	38	2.09	1.00				
Anxiety	None	162	1.98	1.03	2.19	0.07	4 – 309	0.18
Once	49	2.17	1.03				
Twice	38	2.04	0.91				
3–5	27	2.61	1.27				
	6 or more	38	2.10	0.98				
Hostility	None^i^	162	1.91	1.06	2.78	0.03^i^	4 – 309	0.21
	Once^i^	49	1.89	1.07				
	Twice^i^	38	2.35	1.04				
	3–5^i^	27	2.47	1.39				
	6 or more	38	2.24	1.26				

***Source:*** Prepared by the authors based on the study results.

a Symptom Checklist-90-Revised (Derogatis, 1979).

b Possible scores range from 1 to 6; higher scores indicate greater symptom report.

c Total sample may differ by variable due to missing data.

d Mean.

e Standard deviation.

f *P* value for the *F*-test.

g Degrees of freedom.

h Effect size index *f*.

i Significance level *P* < 0.05.

## DISCUSSION

Unlike other studies (14–16), this study did not find an association between risky sexual behavior and HIV status. There seems to be a trend toward condom use during sexual intercourse among both HIV-positive and HIV-negative MSM. However, about 20% of participants reported three or more sexual encounters without condom use. Therefore, it would seem worthwhile to consider whether HIV/AIDS prevention programs are reaching the most vulnerable groups—those living in cities with greater prevalence of HIV, and MSM, according to MINSAL data (2, 3).

The results of this study indicate the need to strengthen messages about the importance of condom use, as the only way to prevent HIV, and as a means of preventing HIV infection and reinfection, in national prevention and selfcare programs for sexually active subjects (15, 16).

These results are consistent with those from other studies that indicate an association between HIV infection and depression symptoms (but not anxiety and hostility symptoms). Some studies (22) have found that depression symptoms in individuals with HIV infection are associated with difficulties in following a treatment. Thus, depression is a variable that should be considered when comprehensively addressing treatment for individuals with this condition.

This study also revealed an association between anxiety symptomatology, feelings of hostility, and risky sexual behavior in MSM. The number of sexual partners in a recent period of time may be related to feelings of anxiety, whereas unprotected sexual relations may be associated with feelings of hostility.

Many studies in other contexts have shown an association between perceived discrimination and maltreatment and various psychological symptoms such as anguish and stress (23, 24). For example, Herek et al. found that victimization experiences due to sexual orientation may be positive predictors of depression symptoms, anxiety, anger, and posttraumatic stress (25). Experiences resulting from violence toward sexual minorities are related to physical and mental symptomatology as well as greater prevalence of risky sexual behaviors (26–28).

A current study conducted in the MSM population in Chile revealed that more than 70% of respondents perceived discrimination due to their sexual orientation or sexual behavior (3). Gómez & Barrientos reported that higher levels of depression symptoms and hostility are related to victimization and discrimination perception (29). In this context, the results show the need to consider the influence of relevant psychosocial stressors, such as the daily discrimination experienced by individuals belonging to sexual minorities (30). Therefore, there is a need to move toward developing social policies that promote a more just and inclusive society, free from discrimination.

Efforts to establish a relationship between psychological symptoms, MSM, risky sexual behavior, and serological status associated with HIV have generated mixed results (6, 8). The possibility that these discrepancies may be due to methodological aspects, such as variable operationalization, focus on symptoms versus diagnosis, and differences in the scales that were used, among others, should be considered (6, 31). These aspects are relevant when establishing links that can lead to the stigmatization of certain groups.

This study did not find differences in sexual risk behaviors between HIV-positive and HIV-negative MSM, but risky sexual behaviors were related to higher levels of anxiety symptoms and hostility. The findings from this study could contribute to better understanding of the risky sexual behaviors of the MSM population in Latin America, and their mental health (32).

### Limitations

This study had some limitations related to certain aspects of its methodology, particularly the population sampling. The target population was sampled with RDS, a methodology that does not allow for random selection of participants, making it more difficult to generalize the findings to the whole population (33, 34). In addition, this type of sample selection introduces bias, which can affect the validity of the results. Therefore, future studies of difficult-to-access populations should use another method of sample selection, such as sampling based on MSM meeting places and/or schedules (35).

Another limitation of this study was the use of self-reported measures. Additional studies on the adaptation of indirect (nonreactive) measurements are needed to determine people’s internal states and attitudes without directly asking about them. These measurement procedures require quicker and less conscious appraisals that make it more difficult to adjust the responses to expectations.

Other limitations were related to the size of the groups studied, and the omission of type of sexual relations and gender of partner. It is preferable to deal with HIV groups (individuals with HIV infection and those who are not infected) of similar sizes to enable comparisons that generate data with greater statistical value. It is also important to include the type of sexual relations (anal or non-anal) and the gender of the sexual partner when measuring risky sexual behavior in MSM because both variables are relevant in that population.

### Conclusions

These results reveal the need to strengthen messages about the importance of condom use, as the only way to prevent HIV, and as a means of preventing HIV infection and reinfection, in national prevention and self-care programs for sexually active subjects. More studies are needed in Latin America to advance HIV prevention efforts for the MSM population. The data generated by this study could be used to inform the development of HIV prevention programming strategies and interventions targeting the MSM population in the region.

#### Funding.

This work is supported by National Commission of Scientific Research and Technology project CONICYT-PCHA/Doctorado Nacional/2015-folio #21150018, and National Fund for Scientific and Technological Development project FONDECYT #1110423 (“Homophobia and psychosocial effects in homosexuals’ quality of life in Chile: Toward a comprehensive model”).

#### Disclaimer.

Authors hold sole responsibility for the views expressed in the manuscript, which may not necessarily reflect the opinion or policy of the RPSP/PAJPH or the Pan American Health Organization (PAHO).

## Footnotes

4 Effect size index *w*, Cohen’s *d*, and effect size index *f*.
